# PtABI3 represses the age biomarker gene *PtDAL1* during male cone development in conifer

**DOI:** 10.48130/forres-0025-0021

**Published:** 2025-09-29

**Authors:** Yi-Tong Song, Feng-Yi Li, Xi Chen, Huan-Huan Zhao, Dong-Yue Wang, Jing-Jing Li, Xiao-Han Zhang, Jun-He Yang, Fang-Xu Han, Pei-Yi Wang, Quan Zuo, Qian-Ya Zhu, Hui Zhang, Biao Zhou, Shi-Hui Niu

**Affiliations:** State Key Laboratory of Efficient Production of Forest Resources, National Engineering Research Center of Tree Breeding and Ecological Restoration, College of Biological Sciences and Technology, Beijing Forestry University, Beijing 100083, PR China

**Keywords:** *Pinus tabuliformis*, Age reset, *DAL1*, *ABI3*, Pollen development

## Abstract

The age biomarker gene *PtDAL1* in conifers undergoes expression resetting during pollen maturation, but its regulatory mechanisms remain unclear. This study identifies *PtABI3*, a B3 transcription factor (TF) in *Pinus tabuliformis*, as a key repressor of *PtDAL1*. *PtABI3* exhibits strict spatiotemporal complementarity with *PtDAL1*. Phylogenetic and structural studies revealed *PtABI3* as a gymnosperm homolog of angiosperm *ABI3*, retaining conserved B3 domains critical for DNA binding and transcriptional repression. Ectopic expression of *PtABI3* in *Arabidopsis* recapitulated the classic *ABI3* late-flowering phenotype. Mechanistically, *PtABI3* directly binds the RY motif (CATGCA) within the *PtDAL1* promoter via its B3 domain, as demonstrated by dual-luciferase assays, yeast one-hybrid assays, and electrophoretic mobility shift assays (EMSA). These results revealed that *PtABI3* is an evolutionary conserved transcriptional repressor that silences *PtDAL1* during pollen maturation, providing the first evidence of B3 TFs mediating age timer gene resetting in conifers. This work bridges gymnosperm and angiosperm regulatory paradigms, highlighting ancient mechanisms for gene expression resetting in plants.

## Introduction

Plant developmental phase transitions throughout their life cycle are meticulously orchestrated by intricate genetic programs and epigenetic regulatory networks. The tightly controlled progression from juvenility to maturity (vegetative phase change) and subsequent shift to reproductive growth displays remarkable stability across diverse environmental conditions^[1,2]^. As sessile organisms, plants employ sophisticated temporal memory systems to coordinate morphological architecture, resource allocation, and developmental timing—critical adaptations ensuring reproductive success^[3]^. This temporal regulation assumes particular significance in perennial woody species where distinct juvenile-to-adult developmental strategies require prolonged vegetative growth prior to first flowering^[4,5]^. The molecular mechanisms underlying plant 'memory' of time remain a central question in plant biology.

Angiosperm research has identified two temporal regulation systems: age-dependent flowering controlled by the microRNA *miR156*^[6−8]^ and vernalization-mediated flowering through *FLOWERING LOCUS C* (*FLC*)^[9,10]^. The evolutionarily conserved *miR156*-SPL (SQUAMOSA PROMOTER BINDING PROTEIN-LIKE) module functions as an endogenous age timer, with declining *miR156* levels coupling cell division history^[11,12]^ to developmental transitions via SPL transcription factors^[13−16]^. Concurrently, *FLC* serves as a vernalization memory integrator where prolonged cold induces Polycomb Repressive Complex 2 (PRC2)-mediated H3K27me3 deposition, stably silencing FLC to permit flowering^[17−19]^.

In the adequate operation of these temporal memory systems, generation after generation, lies a fundamental biological imperative—developmental timers must undergo intergenerational resetting in newly formed embryos. The *miR156* target loci undergo chromatin state reprogramming through a conserved juvenility resetting region (JRR) located upstream of the transcription initiation site^[20]^. This regulatory element exhibits open chromatin architecture enriched with active H3K27ac marks during embryogenesis and juvenility, transitioning to a closed state with repressive H3K27me3 marks in mature plants^[20,21]^. DNA hypomethylation facilitates PRC2 recruitment and H3K27me3 accumulation at *miR156* loci, progressively repressing *miR156* expression via cell division-coupled dilution of active chromatin marks^[22]^. Embryonic factor LEAFY COTYLEDON2 (*LEC2*) orchestrates this resetting through dual mechanisms: recruiting SWITCH/SUCROSE NON-FERMENTING (SWI/SNF) chromatin remodelers to maintain JRR accessibility while suppressing PRC2 activity by repressing FIE (FERTILIZATION INDEPENDENT ENDOSPERM) expression^[20]^.

Parallel vernalization memory resetting occurs through embryonic reactivation of FLC via LEC2-mediated chromatin reorganization and ABA signaling^[23,24]^. FRIGIDA (FRI), a scaffold protein that enhances *FLC* expression by recruiting transcriptional machinery, prevents premature flowering^[25]^, while cold-induced VIVIPAROUS1/ABI3-LIKE 1 (VAL1)/VAL2-PRC2 complexes silence FLC through H3K27me3 deposition at cold memory elements (CMEs)^[26]^. Embryonic maturation triggers ABA accumulation, driving *ABI3/ABI5* recruitment to ABA-responsive elements (ABREs) that reset FLC chromatin to an active state^[27]^.

Contrasting with angiosperm systems, gymnosperms employ unique age-regulatory mechanisms. The MADS-box gene *DAL1* (*DEFICIENS-AGAMOUS-LIKE 1*) functions as a conserved age timer in conifers, showing progressive DNA demethylation and age-correlated upregulation to activate reproductive programs^[28]^. The recent identification of *PtDAL1* (Pt6G35050) as a direct activator of *LEAFY* homologs in *P. tabuliformis* highlights its role in converting temporal information into reproductive signals^[29]^. Intriguingly, *DAL1* undergoes transcriptional silencing during pollen and embryo development across conifer species^[29]^, mirroring *miR156* resetting patterns in angiosperms. However, the mechanistic basis for conifer age-timer resetting remains unknown.

Emerging evidence suggests potential conservation of B3 domain transcription factors (TFs) in developmental timer regulation. Angiosperm B3 proteins (LEC2, ABI3, VAL1) coordinate chromatin resetting through sequence-specific DNA binding^[24,26,27]^, raising intriguing questions about their homologs in gymnosperms. Here, the identification of a B3 family transcription factor, *PtABI3,* is reported as a pivotal regulator of *PtDAL1* silencing during pollen maturation in *P. tabuliformis*. Phylogenetic and molecular analyses characterize *PtABI3* (PtXG06020) as a functional ortholog of angiosperm ABI3. These findings reveal that age-timer regulation is a conserved mechanism, establishing an evolutionary link between angiosperms and gymnosperms in temporal and developmental memory.

## Materials and methods

### Plant materials

#### Plant cultivation and growth conditions

*Arabidopsis thaliana*: plants were grown in a 2:1 mixture of charcoal-enriched and nutrient-supplemented soil at 23 °C under either long-day (16-h light/8-h dark) or short-day (8-h light/16-h dark) photoperiods, with a photosynthetic photon flux density of 120 µmol/m^2^/s.

*Pinus tabuliformis*: samples were collected from a clonal seed orchard in Pingquan City, Hebei Province, PR China, including apical buds, vegetative buds, needles, cambium, roots, female and male cones, pollen, embryos, and ovules. Male cone samples were also collected from mature trees at Beijing Forestry University, China. All samples were immediately flash-frozen in liquid nitrogen and stored at −80 °C. For each tissue type, at least three biological replicates were prepared.

*Nicotiana benthamiana*: plants were grown in a 1:1 mix of vermiculite and charcoal-based soil at 23 °C under long-day conditions (16-h light/8-h dark) with a photosynthetic photon flux density of 120 µmol/m^2^/s. Four-week-old plants with six to eight fully expanded true leaves were used for experiments.

#### RNA-seq analysis

Total RNA was extracted from shoot apex tissues of *Pinus tabuliformis* at various developmental stages using the Plant Total RNA Extraction Kit (Cat. No. RC401-01, Vazyme Biotech Co., Ltd, Nanjing, PR China), following the manufacturer's protocol. To eliminate potential genomic DNA contamination, an on-column DNase I digestion step was included during RNA purification. RNA quantity and purity were assessed using a NanoDrop spectrophotometer (Thermo Fisher Scientific, USA), and RNA integrity was further evaluated using an Agilent 2100 Bioanalyzer (Agilent Technologies, USA). Only RNA samples with an RNA Integrity Number (RIN) > 7.0 were used for subsequent cDNA library construction and RNA-seq analysis.

mRNA was fragmented into shorter sequences through divalent ion-dependent cleavage under elevated temperature conditions. The fragmented RNA was then used for cDNA library preparation via reverse transcription, following the protocol of the Illumina mRNA-Seq Sample Preparation Kit (Illumina, Inc., San Diego, CA, USA). The constructed libraries were subsequently sequenced using paired-end sequencing (2 × 150 bp) on the Illumina NovaSeq platform. After quality filtering, clean reads from each sample were mapped to the *P. tabuliformis* reference genome^[30]^, and transcript quantification was performed using Kallisto. All RNA-seq analyses in this study were performed with three or more biological replicates per condition.

#### Vector construction

All vectors and restriction sites used in this study are shown in Supplementary Table S1, and all primers used in the construction of the vectors are shown in Supplementary Table S2.

#### Transformation of A. thaliana

The coding sequence (CDS) of *PtABI3* was amplified via PCR from *Pinus tabuliformis* cDNA and subsequently cloned into the plant expression vector pBI121 under the control of the 35S promoter. The vector's native *nptII* marker was kept for transgenic event selection. All constructed plasmids were confirmed by Sanger sequencing to verify proper insert orientation and sequence integrity. For *A. thaliana* (ecotype Col-0) transformation, *Agrobacterium tumefaciens* strain GV3101 carrying the expression constructs was employed using the floral dip method^[31]^. Primary transformants were selected on Murashige and Skoog (MS) medium supplemented with 50 mg/L kanamycin. Transgenic seedlings with four to five true leaves were transferred to pots containing a 2:1 mixture of nutrient soil and peat moss. Both transgenic and wild-type control plants were maintained in controlled environment chambers under long-day (LD) conditions.

#### Dual-luciferase reporter assay

The full-length coding sequence (CDS) of *PtABI3*, including its PtABI3_A1B1B2 and PtABI3_B3 domains, was cloned into the pGreen62-SK vector to generate effector constructs. For reporter constructs, the promoter region of *PtDAL1* (–2,301 to 0 bp upstream of the transcription start site) was amplified from genomic DNA and cloned into the pGreenII 0800-LUC vector. A series of *PtDAL1* promoter truncations were generated: *PtDAL1*-*1* (–2,301 to –1,478 bp), *PtDAL1*-*2* (–1,479 to –1,171 bp), *PtDAL1*-*3* (–1,172 to –760 bp), *PtDAL1*-*4* (–761 to –622 bp), *PtDAL1*-*5* (–621 to 0 bp). All recombinant vectors, along with the corresponding negative controls, were transformed into *A. tumefaciens* strain GV3101 harboring the helper plasmid pSoup. Transformed *Agrobacterium* colonies were cultured in LB medium supplemented with appropriate antibiotics. Bacterial cells were harvested by centrifugation at 4,500 rpm for 10 min and resuspended in infiltration buffer (10 mM MES, 10 mM MgCl_2_, 0.2 mM acetosyringone, pH 5.6) to an OD_600_ of ~1.0^[32]^. For *N. benthamiana* infiltration, leaves from four-week-old plants were co-infiltrated with mixtures of effector and reporter constructs. For *P. tabuliformis*, the callus collected at ~14 d post-inoculation was used. After infiltration, plants were kept in the dark for 12 h and then transferred to growth chambers under a 16 h light/8 h dark photoperiod for 48–60 h. Prior to luminescence imaging, leaves were sprayed with 10 μM d-luciferin, and luminescence was captured using a Tianneng5200 multimolecular imaging system. All primers used in this study are listed in Supplementary Table S2.

#### Yeast one-hybrid assay

The A1B1B2 and B3 domains of *PtABI3* were individually cloned into the pB42AD vector using seamless cloning technology, generating the effector constructs pB42AD-PtABI3_A1B1B2 and pB42AD-PtABI3_B3. The *PtDAL1*-*4* promoter fragment (–761 to –622 bp) was inserted into the pLacz2µ vector to create the reporter plasmid pLacZ2µ-*PtDAL1*-*4*. For interaction analysis, both fusion plasmids were co-transformed into the yeast strain EGY48. Four stringent negative controls were established: pB42AD + pLacz2µ, pB42AD + pLacZ2µ-*PtDAL1*-*4*, pB42AD-PtABI3_A1B1B2 + pLacz2µ, pB42AD-PtABI3_B3 + pLacz2µ. Transformants were selected on SD/-Trp/-Ura solid medium and incubated at 28–30 °C for 3–5 d. Six positive colonies were subsequently cultured in liquid SD/-Trp/-Ura medium at 28 °C with 200 rpm shaking for 24–36 h. For *β*-galactosidase assays, 2 μL aliquots of each culture were spotted onto synthetic glucose plates lacking uracil and tryptophan but supplemented with 20 μg/mL X-gal^[33]^. Plates were incubated in darkness for 3–5 d to monitor colony color development.

#### Electrophoretic mobility shift assay

The PtABI3_B3 coding sequence was cloned into the pGEX-4T-1 vector to generate a GST-tagged fusion protein (GST-*PtABI3_B3*), which was expressed in *Escherichia coli* BL21(DE3) cells. Protein expression was induced with 0.3 mM IPTG at 16 °C for 16–18 h after reaching an OD_600_ of 0.8 at 37 °C. For EMSA, biotin-labeled probes (Supplementary Table S3) were prepared using the Biotin Labeling Kit (GS008, Beyotime, PR China). Unlabeled specific probes served as cold competitors, while mutant probes containing 'AAAAAA' substitutions in the RY motifs were used as controls. The EMSA was carried out using the Chemiluminescent Kit (GS009, Beyotime, PR China), and signals were detected on nylon membranes using a BIORAD ChemiDoc™ MP Imaging System. Primer sequences used for EMSA are provided in Supplementary Table S2.

#### Subcellular localization analysis

The 35S::*PtABI3*-GFP construct was generated and transformed into *A. tumefaciens* strain GV3101. Transformed cells were cultured in LB liquid medium containing 50 mg/L kanamycin (Kana) and 50 mg/L rifampicin (Rif) at 28 °C with shaking (200–220 rpm) for 24 h. Bacterial cells were harvested by centrifugation (4,500 rpm, 5 min, room temperature), washed once or twice with infiltration buffer (10 mM MES, pH 5.6, 10 mM MgCl_2_, 150 μM acetosyringone), and resuspended in the same buffer to an OD_6__0__0_ of 0.8–1.2. Suspensions were incubated in the dark at room temperature for 1–3 h before infiltration. For leaf infiltration, bacterial suspensions were pressure-injected into the abaxial epidermis of *N. benthamiana* leaves using a 1 mL syringe. After infiltration, plants were kept in darkness for at least 8 h, then transferred to a 16 h light/8 h photoperiod for 48–60 h. GFP fluorescence was visualized using a Leica SP8 confocal microscope with 488 nm excitation and emission detection at 505–530 nm.

#### Phylogenetic analyses

To analyse the phylogenetic relationships of *ABI3* homologs in *P.* *tabuliformis* with those from other plants, a phylogenetic tree was constructed. The amino acid sequences of *ABI3* homologs from all species (Supplementary Table S4) were aligned using the CLUSTAL algorithm. The phylogenetic tree was constructed in MEGA X software using its maximum likelihood (ML) method, with bootstrap values set to 800 replicates. The protein sequences used in this study were downloaded from the GenBank database (www.ncbi.nlm.nih.gov).

### Statistical analyses

All statistical analyses were performed using GraphPad Prism 8. Data are presented as mean ± standard deviation (SD) unless otherwise specified. Statistical significance was determined using Student's t-test depending on the experimental design and data distribution.

Error bars in the figures represent the standard deviation (SD) of the mean. A *p-*value of less than 0.05 was considered statistically significant (* *p* < 0.05), less than 0.01 was considered a highly significant difference (** *p* < 0.01), and less than 0.001 was considered an extremely significant difference (*** *p* < 0.001).

#### Network construction

Weighted gene co-expression network analysis (WGCNA) was performed on all samples collected during male cone development using standard methodologies^[34,35]^. Genes were hierarchically clustered via average linkage based on topological overlap measures, and a clustering dendrogram was generated. Subsequently, the correlation between hub genes within the module containing *PtDAL1* and male cone developmental stages was systematically evaluated.

#### Protein structure prediction

The three-dimensional structure of PtABI3 was predicted using AlphaFold3^[36]^, the latest iteration of the deep learning-based protein structure prediction system. The full-length amino acid sequence of PtABI3 was submitted to the AlphaFold3 pipeline with default parameters. The model was generated using the multimer prediction mode to account for potential intra-molecular interactions. Five models were produced for each prediction, with the highest-ranked model selected based on the predicted local distance difference test (pLDDT) score. The predicted structure was visualized and analysed using PyMOL Molecular Graphics System (Version 2.5.2, Schrödinger, LLC). Structural domains were identified through comparison with known B3 domain-containing proteins in the Protein Data Bank (PDB).

#### Accession numbers

Sequence data used or produced for this article can be found in the GenBank/EMBL data libraries and CPIR (http://conifers.cn/#/) under the accession numbers in Supplementary Table S4.

## Results

### Identification of transcription factors with expressions correlated to *DAL1* during male cone development in *P. tabuliformis*

**Figure 1:**
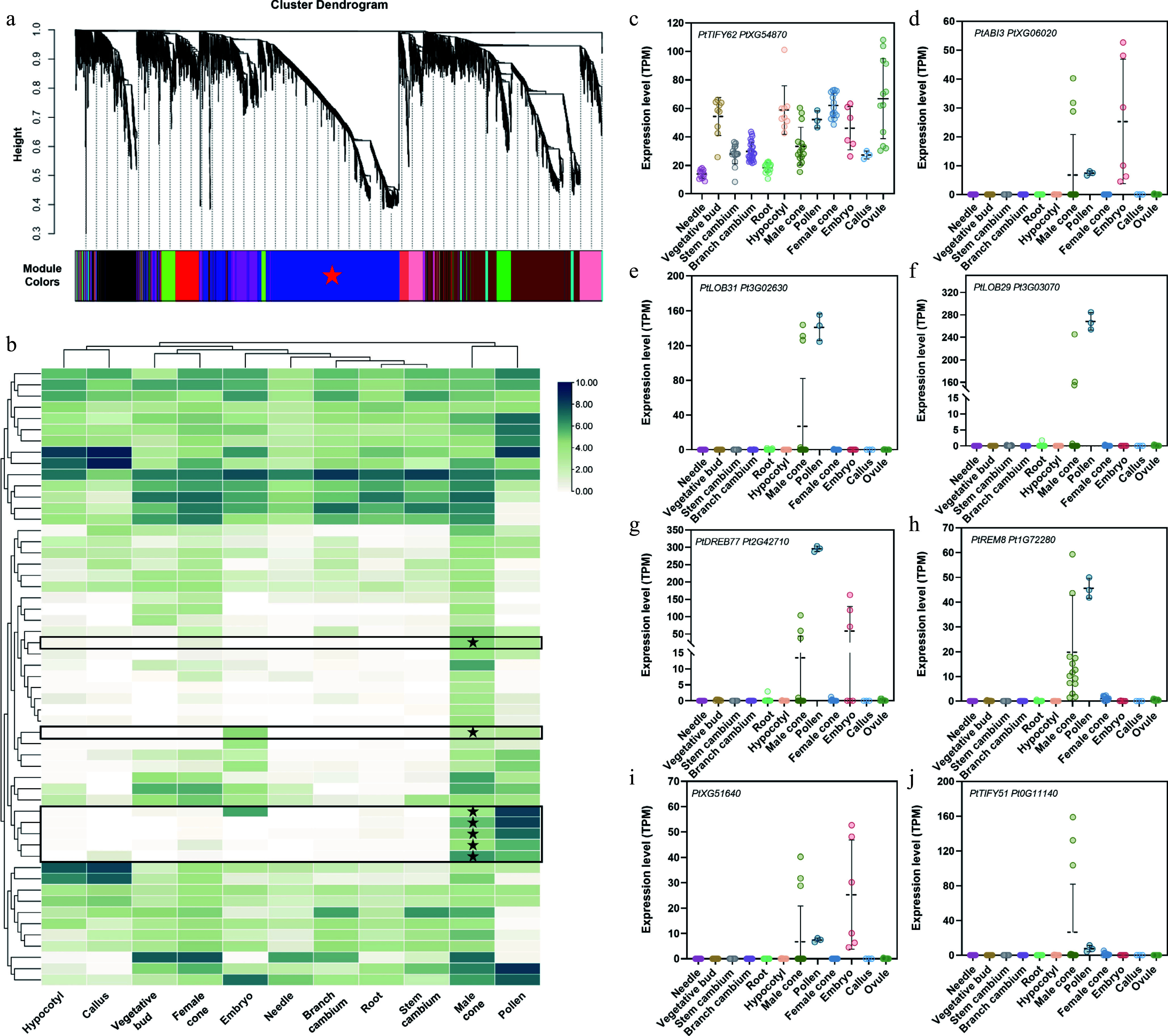
Identification of transcription factors associated with *DAL1* expression during the development of the male cone in *P. tabuliformis*. (a) WGCNA analysis of genes at different developmental stages of the male cone. Cluster dendrograms were obtained using the gene average chain hierarchical clustering method based on topological overlap. Co-expressed gene modules are indicated by different color bars below the dendrogram. (b) Expression of core transcription factors related to the resetting process of *PtDAL1* in male cones in different tissues, where expression has been log-transformed. (c)–(j) Expression of core transcription factors related to the resetting process of *PtDAL1* in the male cone in different tissues.

The previous study revealed that *DAL1* serves as a conserved age biomarker in conifers^[30,37]^. Interestingly, its expression undergoes dramatic suppression during the late stage of male cone development and is completely silenced in mature pollen^[28]^. To investigate regulatory factors involved in this *DAL1* expression resetting process, weighted gene co-expression network analysis (WGCNA) was performed on transcriptomes from five developmental stages of male cones of *P. tabuliformis*. The result revealed 2,979 genes co-clustered with *DAL1* in the blue module (MEblue) (Fig. 1a). Using thresholds of both gene significance (GS) and module membership (kME) greater than 0.9, the candidate list was further refined to 860 genes, including 56 transcription factors (TFs). Tissue-specific expression profiling (Fig. 1b) showed that some genes, such as *PtXG51640* (Fig. 1c), displayed ubiquitous expression across multiple tissues—a pattern inconsistent with *PtDAL1*'s strict resetting specificity to reproductive tissues. Notably, half of the candidates exhibited reproductive tissue-specific expressions (Fig. 1b), as potential *DAL1* resetting regulators, such as *PtABI3*, *PtLOB31*, *PtLOB29*, *PtDREB77*, *PtREM8*, *PtXG51640*, *PtTIFY62*, and *PtTIFY51* (Fig. 1d–j).

### Spatiotemporal complementarity of *PtABI3* and *PtDAL1* expression patterns in different tissues and organs of *P. tabuliformis*

**Figure 2:**
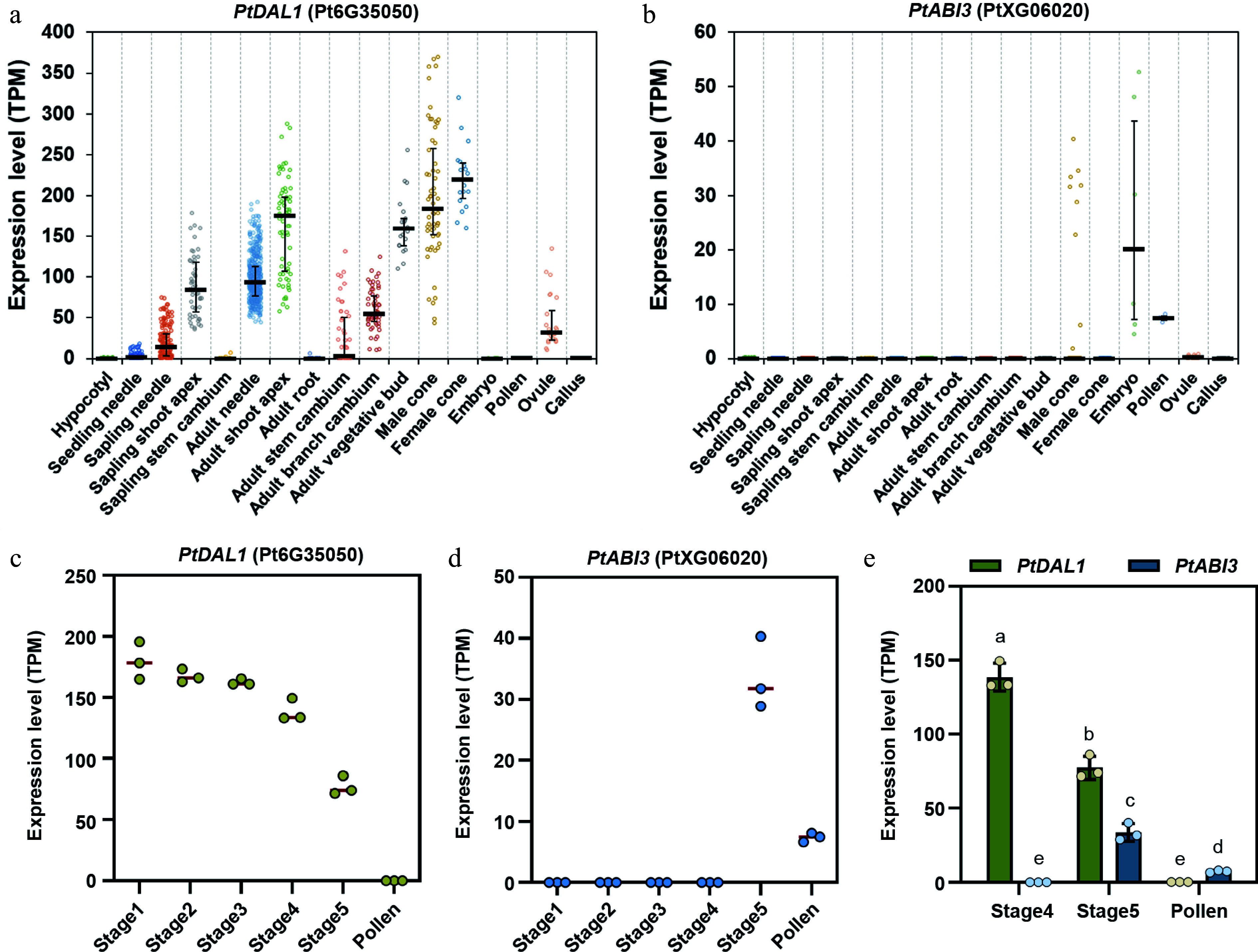
Expression patterns of *PtDAL1* and *PtABI3* in different tissues and developmental stages of *P. tabuliformis*. (a), (b) Expression patterns of *PtDAL1* and *PtABI3* in different tissues of *P. tabuliformis*, showing a clear spatial complementary relationship. (c), (d) Expression patterns of *PtDAL1* and *PtABI3* during pollen development in *P. tabuliformis*, showing a clear temporal complementary relationship^[28]^. (e) Expression patterns of *PtDAL1* and *PtABI3* in the final two stages of male cone development and pollen in *P. tabuliformis*.

Given that B3 family TFs are known to play pivotal roles in resetting expression of the angiosperm age timer *miR156* and vernalization memory factor *FLC*^[29]^, the focus was on *PtABI3*, also a B3 family member in *P. tabuliformis.* Intriguingly, *PtABI3* exhibits striking spatiotemporal complementarity with *PtDAL1* expression in different tissues and organs across developmental stages. *PtABI3* is exclusively expressed in male cones, mature pollen, and embryos, whereas *PtDAL1* shows suppressed or undetectable expression in these tissues (Fig. 2). During late stages of male cone development, a stage-specific expression switch was observed (Fig. 2c*–*e). At stage four (April 12) of male cone development, *PtABI3* remained silent while *PtDAL1* showed high expression. This pattern dramatically reversed at stage five (April 20), with *PtABI3* abruptly activated and *PtDAL1* expression halved (Fig. 2c–e). The two genes exhibited a strong negative correlation (Pearson's *R* = –0.96). Notably, *PtDAL1* was completely silenced in mature pollen, where *PtABI3* remained active (Fig. 2d, e). This inverse temporal-spatial regulation suggests *PtABI3* may directly participate in resetting *PtDAL1* expression during reproductive development, mirroring B3 TFs' conserved silencing mechanisms in flowering plants.

### *PtABI3* encodes a B3 transcription factor with a conserved DNA-binding domain

**Figure 3:**
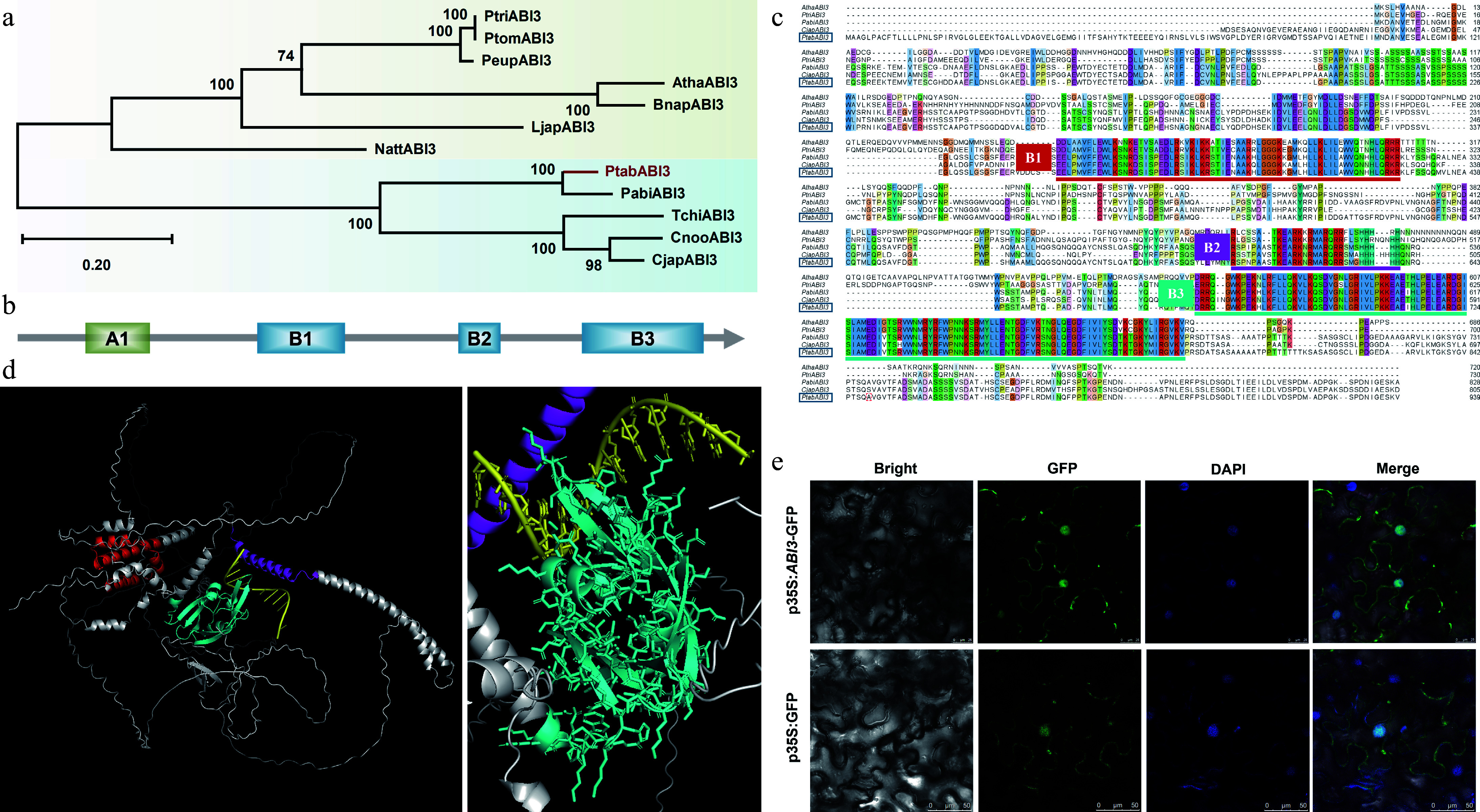
Phylogenetic and structural analysis of PtABI3. (a) The phylogenetic tree of PtABI3, with the species and sequence information used presented in Supplementary Table S4. PtABI3 is highlighted in red in the phylogenetic tree. (b) Schematic diagram of the conserved domains in the PtABI3 protein, including four conserved domains: A1, B1, B2, and B3. (c) Multiple sequence alignment of the amino acid sequences of the ABI3 proteins, with conserved domains marked by horizontal lines of different colors. The PtABI3 sequence is highlighted with a blue box. (d) Three-dimensional structure prediction of PtABI3, where red represents the B1 domain, purple represents the B2 domain, and cyan represents the B3 domain. The right figure is a magnified view of the left figure, showing the binding pattern of PtABI3 with the DNA sequence. (e) Subcellular localization of the PtABI3 protein in *N. benthamiana* epidermal cells.

Phylogenetic analysis showed that PtABI3 is a homolog of angiosperm ABI3 proteins (Fig. 3a). The PtABI3 protein, like its homologs, comprises four conserved domains: A1, B1, B2, and B3 (Fig. 3b), which are critical for transcriptional regulation (A1), nuclear localization and protein interactions (B1, B2), as well as DNA binding (B3)^[38,39]^. Although gymnosperm ABI3 proteins and angiosperm homologs form two distinct clades (Fig. 3a), their core domains remain highly conserved, particularly the B1, B2, and B3 domains (Fig. 3c). AlphaFold structural predictions indicate that the B3 domain of PtABI3 serves as the primary DNA-binding domain (Fig. 3d), with its sequence and structure being remarkably conserved across seed plants (Fig. 3c; Supplementary Fig. S1). This conservation suggests not only its indispensable functional role but also potentially shared DNA motif recognition mechanisms during evolution.

Subcellular localization assays in tobacco leaf epidermal cells demonstrated nuclear enrichment of PtABI3, with additional cytoplasmic distribution (Fig. 3e), consistent with its role in transcriptional regulation. Collectively, these findings imply that PtABI3 likely retains transcriptional regulatory functions analogous to its angiosperm counterparts.

### Overexpression of *PtABI3* causes late flowering in *Arabidopsis*

**Figure 4:**
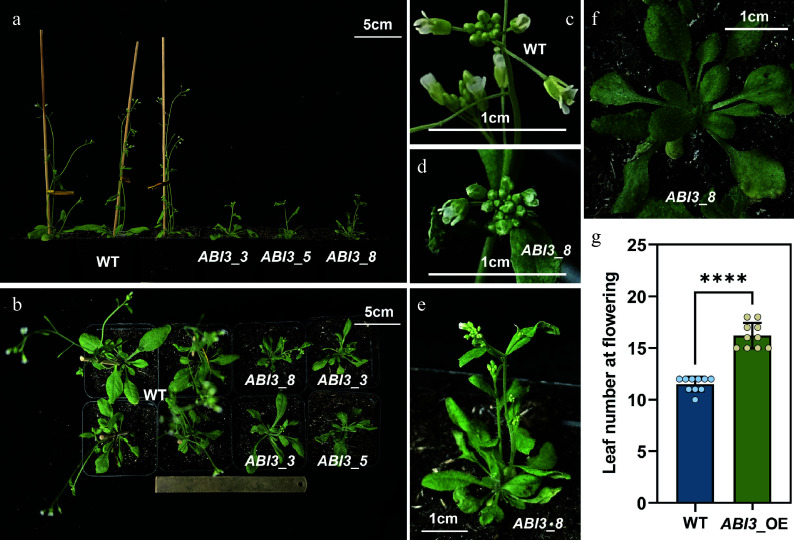
Overexpression of *PtABI3* in *A. thaliana*. (a), (b) Compared to wild-type (WT) *Arabidopsis*, all 35S::*PtABI3* lines exhibit a distinct late-flowering and delayed-development phenotype. (c), (d) Representative images of inflorescences from 35S::*PtABI3*_8 lines and WT *Arabidopsis*. (e), (f) Images of 35S::*PtABI3*_8 lines during development, showing the number of rosette leaves immediately before and at the bolting stage. (g) The number of rosette leaves was counted at the first flowering stage for both wild-type and 35S::*PtABI3* lines, **** *p* < 0.0001.

Given the current technical challenges in genetic transformation of *P. tabuliformis*, which include low transformation efficiency and recalcitrance of tissues to regenerate, functional validation of *PtABI3* was conducted in the model plant *A. thaliana*. Overexpressing *PtABI3* plants exhibited distinct morphological alterations compared to wild-type (WT) *Arabidopsis* (Fig. 4). The most conspicuous phenotype was significantly delayed flowering time (Fig. 4a, b). Concurrently, 35S::*PtABI3* lines produced more inflorescences than WT (Fig. 4c, d), along with overall plant dwarfism and reduced leaf size (Fig. 4b, e). Remarkably, 35S::*PtABI3* plants persisted in vegetative growth when WT plants bolted, initiating reproductive growth only after developing 15–18 leaves (Fig. 4f, g). These phenotypic similarities mirror observations in angiosperm *PtABI3*-overexpression lines, suggesting potential overlap in downstream target gene sets between *PtABI3* and its angiosperm counterparts in *Arabidopsis*.

### *PtABI3* represses *PtDAL1* transcription via direct binding to the RY motif in its promoter

**Figure 5:**
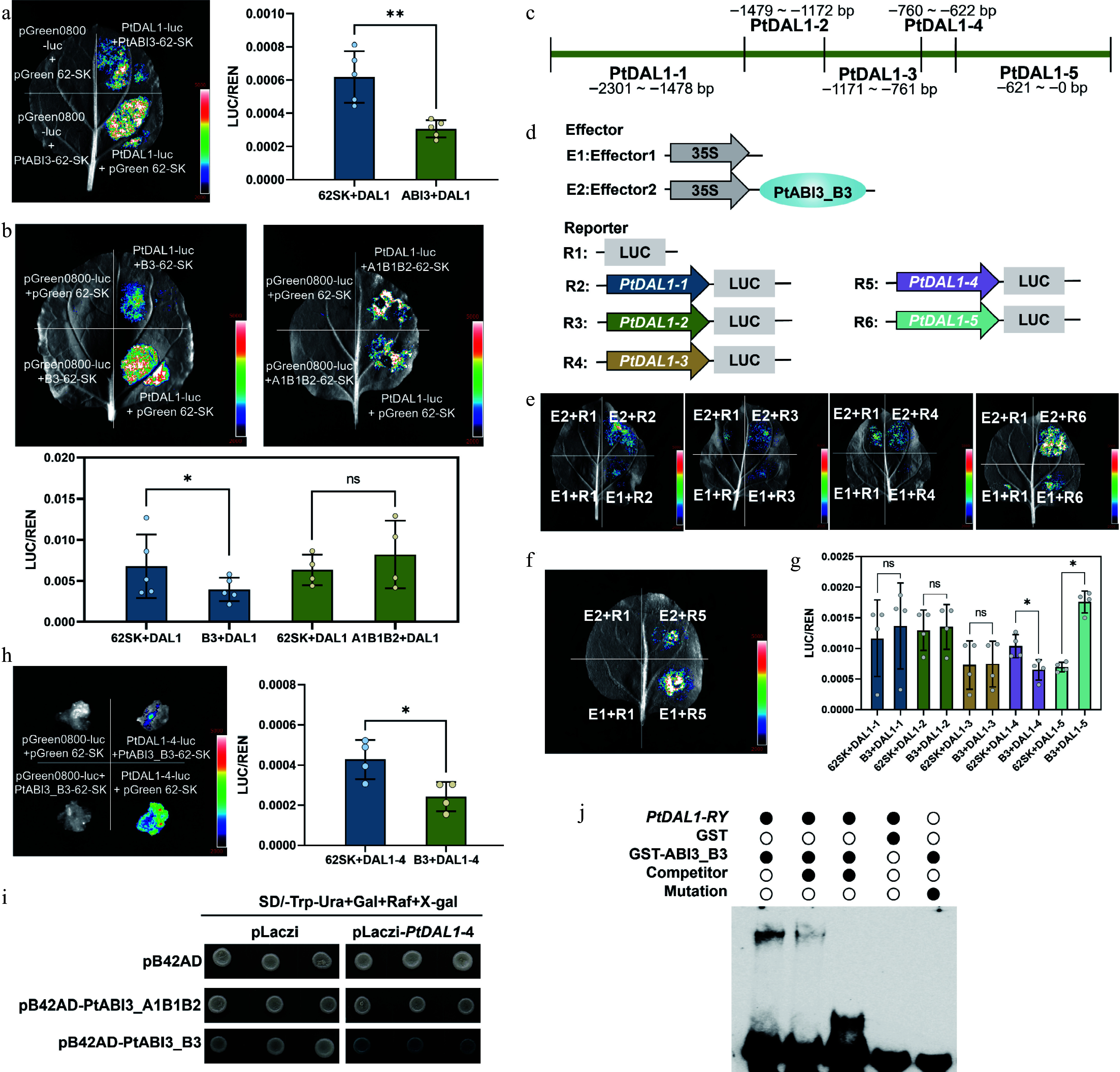
PtABI3 binds directly to the RY motif in the *PtDAL1* promoter region to inhibit its expression. (a) A dual luciferase reporter assay was performed on the full-length PtABI3 protein and the *PtDAL1* promoter. Differential analysis indicated that PtABI3 significantly inhibits the *PtDAL1* promoter (**, *p* < 0.01). (b) Dual luciferase reporter assays were performed using different domains of PtABI3 and the *PtDAL1* promoter. (*, *p* < 0.05, ns, *p* > 0.05). (c) Schematic diagram of the truncation sites in the *PtDAL1* promoter region. (d) Schematic diagram of vector construction. (e), (f) Dual luciferase reporter assay of PtABI3_B3 and five *PtDAL1* promoter fragments. (g) Quantitative detection of dual luciferase assay LUC (firefly *luciferase*) fluorescence values. (h) Dual luciferase assay of PtABI3_B3 and *PtDAL1*-*4* in *P. tabuliformis* callus. (i) Direct binding of PtABI3_B3 to the *PtDAL1*-*4* promoter in a yeast one-hybrid system. (j) EMSA using a biotin-labeled DNA probe containing the RY motif from *PtDAL1*-*4* and purified GST-PtABI3_B3 protein. GST protein, unlabeled probes (competitors), and mutant probes were used as controls.

To determine whether PtABI3 directly regulates *PtDAL1* expression, dual-luciferase reporter (DLR) assays were performed using a –2,301 bp fragment of the *PtDAL1* promoter region. The result showed that PtABI3 significantly suppressed *PtDAL1* promoter activity (Fig. 5a). Given that *PtABI3* contains four structural domains (Fig. 3b), truncations retaining either the A1B1B2 domains or the B3 domain were generated to identify the functional domain responsible for this repression. Only PtABI3_B3 exhibited significant inhibitory effects (Fig. 5b), indicating the indispensability of the B3 domain.

To pinpoint the *PtDAL1* promoter region bound by PtABI3, the –2,301 bp of the *PtDAL1* promoter region was divided into five segments (Fig. 5c). DLR assays revealed that PtABI3_B3 binds to *PtDAL1*-4 (–761 to –621 bp) and *PtDAL1*-5 (–621 to –1 bp) but only repress transcriptional activation mediated by *PtDAL1*-4 (Fig. 5d–g). Notably, the binding of PtABI3 to the *PtDAL1*-5 segment exhibits a certain level of promoter activity; however, the net repressive effect observed on the full-length promoter indicates that the inhibitory effect mediated by *PtDAL1*-4 is the dominant regulatory mechanism. To validate this regulation in the native system, the experiment in *P. tabuliformis* callus was replicated, confirming that PtABI3_B3 suppresses *PtDAL1* promoter activity in its endogenous context (Fig. 5h). Yeast one-hybrid assays further demonstrated direct binding of PtABI3_B3 to segment four of *PtDAL1* promoter (Fig. 5i). Notably, segment four contains an RY motif (CATGCA, –649 to –644 bp), a conserved binding site for B3 domain proteins. Electrophoretic mobility shift assays (EMSA) using a 39-bp biotin-labeled probe spanning the RY motif (–663 to –624 bp) showed that GST-PtABI3_B3 fusion protein specifically binds the wild-type probe but not a mutated RY motif control (Fig. 5j). These results indicate that PtABI3 directly represses *PtDAL1* expression in *P. tabuliformis* by binding the RY motif in its promoter via the B3 domain. This mechanism may serve as a critical component of *PtDAL1* expression resetting during pollen maturation.

## Discussion

**Figure 6:**
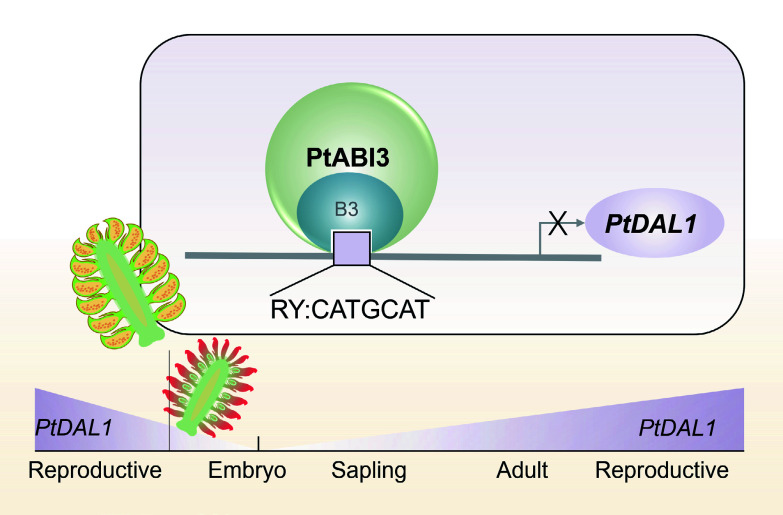
Schematic diagram of PtABI3 inhibition of *PtDAL1* during male gametophyte development. The age biomarker *DAL1* is reset during intergenerational alternation. During male gametophyte development, PtABI3 directly binds to the RY motif on the *PtDAL1* promoter through the B3 domain, directly inhibiting its expression.

As a representative conifer species, *P. tabuliformis* undergoes complex and tightly regulated developmental transitions during its reproductive cycle. This regulation is governed by an age-dependent molecular network that ensures proper timing and coordination of key developmental events. In this study, *PtABI3* (PtXG06020) was identified as a central regulator that directly suppresses the expression of *PtDAL1* (Pt6G35050), the age bio-marker, through binding to RY cis-regulatory elements (CATGCA). Moreover, a functional model was proposed wherein PtABI3 mediates the resetting of the age-associated marker *PtDAL1* during male gametophyte development (Fig. 6). These findings expand the understanding of ABI3 family functions in gymnosperms and provide novel insights into the evolution of regulatory mechanisms underlying reproductive phase transitions in seed plants.

In angiosperms, B3-domain-containing transcription factors such as VAL (VP1/ABI3-LIKE) are known to repress embryonic gene programs by recognizing Sph/RY motifs (CATGCA), thereby preserving meristematic identity^[40]^. The RY motif is a highly conserved cis-element central to seed development, serving as the binding site for B3 domain proteins^[41]^. It is pivotal for the expression of seed storage protein genes and the establishment of dormancy^[42,43]^. Beyond this, it is implicated in stress responses^[44]^, flowering time regulation^[45]^, and, as this work shows, in vegetative developmental transitions in conifers. Given the high conservation of the B3 domain across seed plants (Fig. 3c), it is reasonable to hypothesize that similar regulatory mechanisms may operate in gymnosperms. These results confirm that PtABI3 specifically binds to the RY motif within the *PtDAL1* promoter via its B3 domain, leading to direct transcriptional repression. This mechanism shares temporal features with the ABI3-*FLC* regulatory module in *Arabidopsis*^[29]^. However, there are differences in the regulatory directions; Arabidopsis ABI3 acts synergistically with *LEC1* (LEAFY COTYLEDON 1) to erase vernalization memory and reactivate *FLC* (*FLOWERING LOCUS C*) expression, while PtABI3 inhibits *PtDAL1*. This divergence may reflect functional specialization driven by evolutionary adaptation in gymnosperms. Please note that our DLR analysis shows that the binding of the *PtDAL1*-5 segment to PtABI3 exhibits a certain level of activation capacity. This suggests the existence of a complex regulatory pattern: the net inhibitory effect of PtABI3 on *PtDAL1* is most likely caused by the dominant inhibitory effect mediated by the *PtDAL1*-4 segment containing the RY motif, which overrides the potential activation effect mediated by other regions such as *PtDAL1*-5. The balance between these inhibitory and activating forces may enable the fine-tuned regulation of *PtDAL1* expression.

*ABI3* exhibits dynamic regulatory roles across developmental stages. For example, in *Arabidopsis*, *ABI3* differentially modulates *miR156* expression. It interacts with various bZIP cofactors (AREB, ABSCISIC ACID-RESPONSIVE ELEMENT BINDING PROTEIN) to form transcriptional complexes that bind ABRE sites, influencing multiple physiological processes^[46−48]^. The dwarfism and delayed flowering observed in our *PtABI3*-OE Arabidopsis lines are consistent with the findings of Zhang et al. in soybean (*Glycine max*)^[49]^. Ectopic expression of a master seed regulator like ABI3 in vegetative tissues likely causes widespread misregulation of gene networks. Additionally, the B2 domain of ABI3/VP1 has been shown to induce nucleosome sliding, promoting open chromatin structures in target promoters^[50]^. A well-characterized case is the regulation of *At2S3*, where FUS3 (FERTILIZATION INDEPENDENT SEED DEVELOPMENT 3) and LEC2 directly bind RY elements. At the same time, ABI3 cooperates with bZIP proteins bound to G-box motifs (CACGTG), forming a 'dual-factor synergistic enhancer' that amplifies gene expression signals^[51]^. Therefore, future studies should investigate whether PtABI3 employs similar protein interaction strategies to regulate *PtDAL1* expression during male cone development.

Despite growing interest in conifer developmental biology, research on *ABI3* homologs remains limited. Evidence from heterologous expression experiments indicates that *ABI3* from *Chamaecyparis nootkatensis* can partially rescue the *abi3* mutant phenotype in *Arabidopsis*^[52]^, suggesting that core functions of *ABI3* are conserved across seed plants. However, the distinct reproductive strategies of gymnosperms imply that *ABI3* may have evolved unique roles in conifers. The PtABI3-mediated regulation of *PtDAL1* expression observed in this study, supported by yeast one-hybrid, EMSA, and transient dual-luciferase assays in callus tissue, provides molecular evidence supporting a potential age biomarker resetting mechanism in gymnosperm reproductive development (Fig. 6). These findings may contribute to understanding the evolution of regulatory networks in ancestral seed plants.

While these findings represent significant progress, several questions remain unresolved. First, the precise regulatory position of PtABI3 within the *PtDAL1* resetting pathway is still unclear, and the identities of potential interacting partners or upstream regulators remain to be determined. Second, although the current analysis focuses on male reproductive tissues, future work should assess whether a similar resetting mechanism operates in female reproductive organs. Finally, the involvement of epigenetic modifications, histone remodeling, or non-coding RNA pathways in the clearance of age-associated markers warrants further investigation.

## Conclusions

This work provides the first evidence that B3 transcription factors mediate age-biomarker *PtDAL1* expression resetting in conifers, bridging a fundamental gap between gymnosperm and angiosperm regulatory paradigms.

This study demonstrates that PtABI3 is a gymnosperm ortholog of angiosperm ABI3 proteins, retaining conserved functional domains and regulatory properties. PtABI3 appears to silence *PtDAL1* during pollen maturation, likely through B3 domain-mediated binding to the RY motif in its promoter. This repression mechanism exhibits striking parallels with B3 TF function in angiosperms, suggesting an ancient origin for transcriptional resetting of developmental timing genes in seed plants.

These findings not only advance understanding of conifer reproductive biology but also reveal a conserved regulatory mechanism, reflecting the deep conservation of transcriptional regulation across 300 million years of seed plant evolution. Future studies should explore whether this PtABI3*-PtDAL1* module coordinates with other age-related pathways to govern developmental transitions in perennial conifers.

## SUPPLEMENTARY DATA

Supplementary data to this article can be found online.

## Data Availability

The datasets generated during and/or analyzed during the current study are available from the corresponding author on reasonable request. The RNA-seq data that support the findings of this study have been deposited in CPIR (http://conifers.cn/).
