# Supplementary material for: PtABI3 represses the age biomarker gene PtDAL1 during male cone development in conifer
Source: For Res (Fayettev). 2025 Sep 29;5:e021. doi: 10.48130/forres-0025-0021 (PMC12569429; doi:10.48130/forres-0025-0021)
Supplement: Supplementary file 1 — Supplementary data to this article can be found online. [file FR-2025-5-0021-Supplementary.zip › 10.48130_forres-0025-0021-Suppl-TableS1.pdf]

Table S1 Selection of linearization digest sites for seamless cloning vectors.

Vectors	Restriction Endonucleases
pB42AD	*Xho*I
pLacZ-2μ	*Xho*I
pGreen0800-Luc	*Xho*I+*Bam*HI
pGreen62-SK	*Eco*RI
pGEX4T-1	*Xho*I
pBI121-GFP	*Kpn*I+*Xba*I
pCambia1300-GFP	*Sac*I+ *Bam*HI
pGADT7	*Eco*RI+*Bam*HI
pGBKT7	*Eco*RI+*Bam*HI
