# Supplementary material for: PtABI3 represses the age biomarker gene PtDAL1 during male cone development in conifer
Source: For Res (Fayettev). 2025 Sep 29;5:e021. doi: 10.48130/forres-0025-0021 (PMC12569429; doi:10.48130/forres-0025-0021)
Supplement: Supplementary file 1 — Supplementary data to this article can be found online. [file FR-2025-5-0021-Supplementary.zip › 10.48130_forres-0025-0021-Suppl-TableS2.pdf]

Table S2 Primer information used to construct vectors in this study.

Primer name	Primer sequence (5'-3')
	pB42AD
PtABI3 -42AD-F	cccgaattcggccgactcgagatggctgcgggcttgcct
PtABI3-42AD-R	agaagtccaaagcttctcgagaactttgctttctccaatattatcagg
PtABI3_B3-42AD-F	cccgaattcggccgactcgaggaatacatgaactaccgctctcca
PtABI3_B3-42AD-R	agaagtccaaagcttctcgagaactttgctttctccaatattatcagg
PtABI3_A1B1B2-42AD-F	cccgaattcggccgactcgagatggctgcgggcttgcct
PtABI3_A1B1B2-42AD-R	agaagtccaaagcttctcgagtaaataggattgtgaagatgcaaagc
	pLacZ-2μ
PtDAL1-LACZ-F	atctgtcgacctcgagttgaagattttatgaagtgagagga
PtDAL1-LACZ-R	gagcacatgcctcgagtcattgagtcaacgtttgatatact
	pGreen0800-Luc
PtDAL1-0800-F	ggtaccgggccccccctcgagaatgagttctaaatttcatctatgtctgat
PtDAL1-0800-R	cgctctagaactagtggatcctgcaagatccctctgcttcttc
PtDAL1_1-0800-F	gaatcatttgggtcatgcatcctatgcatgctcatcgat
PtDAL1_1-0800-R	atcgatgagcatgcataggatgcatgacccaaatgattc
PtDAL1_2-0800-F	ggtaccgggccccccctcgagaatccatgtggaattcacgtagatt
PtDAL1_2-0800-R	cgctctagaactagtggatccactgccaaaccaaaggcgt
PtDAL1_3-0800-F	ggtaccgggccccccctcgagtgtggacacgcctttggttt
PtDAL1_3-0800-R	cgctctagaactagtggatccacaaacaaggcagtggaaatgc
PtDAL1_4-0800-F	ggtaccgggccccccctcgagcgatgatgtatgggcatgtgg
PtDAL1_4-0800-R	cgctctagaactagtggatcccattccatataaagtaaaccaagcca
PtDAL1_5-0800-F	ggtaccgggccccccctcgagggcgaaaggcatgtgatgg
PtDAL1_5-0800-R	cgctctagaactagtggatccttatcgatgagcatgcataggatg
	pGreen62-SK
PtABI3-62SK-F	tcccccgggctgcaggaattcatggctgcgggcttgcct
PtABI3-62SK-R	gataagcttgatatcgaattcaactttgctttctccaatattatcagg
PtABI3_B3-62SK-F	tcccccgggctgcaggaattcgaatacatgaactaccgctctcca
PtABI3_B3-62SK-R	gataagcttgatatcgaattcaactttgctttctccaatattatcagg
PtABI3_A1B1B2-62SK-F	tcccccgggctgcaggaattcatggctgcgggcttgcct
PtABI3_A1B1B2-62SK-R	gataagcttgatatcgaattctaaataggattgtgaagatgcaaagc
	pGEX4T-1
PtABI3_B3-GST-F	gaattcccgggtcgactcgaggaatacatgaactaccgctctcca
PtABI3_B3-GST-R	gtcacgatgcggccgctcgagaactttgctttctccaatattatcagg
	pBI121-GFP
PtABI3-121-F	gcccttgctcaccatggtaccatggctgcgggcttgcct
PtABI3-121-R	gagaacacgggggactctagaaactttgctttctccaatattatcagg
