# Supplementary material for: PtABI3 represses the age biomarker gene PtDAL1 during male cone development in conifer
Source: For Res (Fayettev). 2025 Sep 29;5:e021. doi: 10.48130/forres-0025-0021 (PMC12569429; doi:10.48130/forres-0025-0021)
Supplement: Supplementary file 1 — Supplementary data to this article can be found online. [file FR-2025-5-0021-Supplementary.zip › 10.48130_forres-0025-0021-Suppl-TableS3.pdf]

Table S3 EMSA probe sequences used in this section.

**Name**	**5'-labeled TAMRA**
PtDAL1-RY	gaatcatttgggt**catgca**tcctatgcatgctcatcgat
Mutation	gaatcatttgggt**aaaaaa**tcctatgcatgctcatcgat

The table shows the probe sequences used in the EMSA experiments in this study, with the RY motif and mutation sites highlighted in red bold font.
