# Supplementary material for: PtABI3 represses the age biomarker gene PtDAL1 during male cone development in conifer
Source: For Res (Fayettev). 2025 Sep 29;5:e021. doi: 10.48130/forres-0025-0021 (PMC12569429; doi:10.48130/forres-0025-0021)
Supplement: Supplementary file 1 — Supplementary data to this article can be found online. [file FR-2025-5-0021-Supplementary.zip › 10.48130_forres-0025-0021-Suppl-TableS4.pdf]

Table S4 Protein sequence information used in phylogenetic analysis and multiple sequence alignment.

Species	Protein	Accession Number
Populus trichocarpa	PtriABI3	CAA05921.1
Populus tomentosa	PtomABI3	AGM20671.1
Populus euphratica	PeupABI3	XP_011024550.1
Arabidopsis thaliana	AthaABI3	AT3G24650.1
Brassica napus	BnapABI3	NP_001302690.1
Lotus japonicus	LjapABI3	XP_057440479.1
Nicotiana attenuata	NattABI3	XP_019238002.1
Pinus tabuliformis	PtabABI3	
Picea abies	PabiABI3	AAG22585.1
Taxus chinensis	TchiABI3	KAH9314889.1
Callitropsis nootkatensis	CnooABI3	CAC19186.1
Cryptomeria japonica	CjapABI3	XP_057858141.2
