# Supplementary material for: PtABI3 represses the age biomarker gene PtDAL1 during male cone development in conifer
Source: For Res (Fayettev). 2025 Sep 29;5:e021. doi: 10.48130/forres-0025-0021 (PMC12569429; doi:10.48130/forres-0025-0021)
Supplement: Supplementary file 1 — Supplementary data to this article can be found online. [file FR-2025-5-0021-Supplementary.zip › 10.48130_forres-0025-0021-Suppl-FigureS1.pdf]

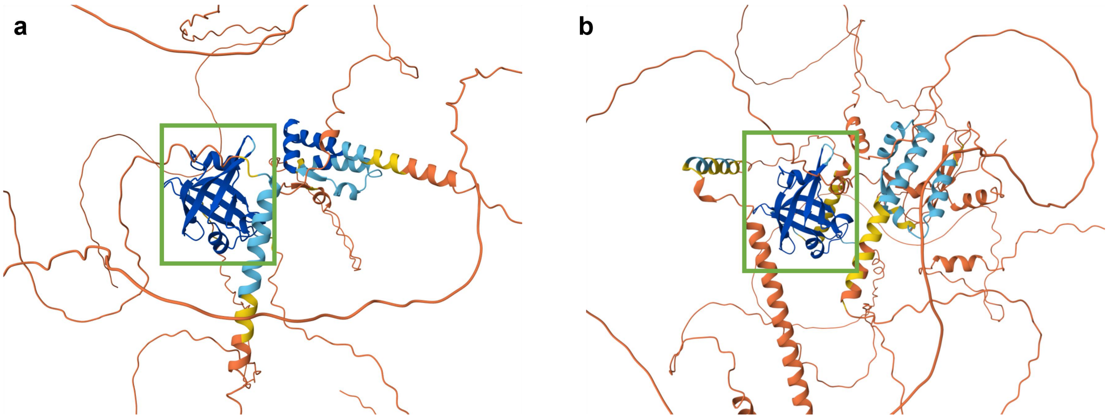

Fig. S1 Comparison diagram of predicted structures of ABI3 proteins.

(a) 3D structure prediction of the ABI3 (AT3G24650.1) protein in *Arabidopsis thaliana*, where the B3 domain is the dark blue structure within the green box. (b) 3D structure prediction of the PtABI3 protein in *P. tabuliformis*, where the B3 domain is the dark blue structure within the green box.
